# Prevalence and associated anthropometric and lifestyle predictors of hypertension among adults in Kombolcha town and suburbs, Northeast Ethiopia: a community-based cross-sectional study

**DOI:** 10.1186/s12872-019-1225-x

**Published:** 2019-10-29

**Authors:** Biniem Hassen, Hassen Mamo

**Affiliations:** Department of Microbial, Cellular and Molecular Biology, College of Natural and Computational Sciences, Addis Ababa University, PO Box 1176, Addis Ababa, Ethiopia

**Keywords:** Hypertension, Anthropometric, Lifestyle, Cross-sectional

## Abstract

**Background:**

Hypertension (HTN) is major public health challenge. Data on HTN prevalence and associated risk factors is necessary to better control it. This study aimed at estimating the prevalence of HTN and associated anthropometric and lifestyle predictors in Kombolcha and suburbs, northeast Ethiopia.

**Methods:**

The study followed a community-based cross-sectional sampling design. Adult (≥18) residents of Kombolcha and suburbs in 11 *kebeles* (villages) formed the target population. Of these *kebeles*, 5(3 urban and 2 suburban) were selected randomly. Households (HHs) within the selected *kebeles* and individuals within HHs were similarly recruited in December 2016–May 2017. Anthropometric and blood pressure measurements were done. World Health Organization (WHO) STEPWISE TOOL was used to capture socio-demographic, physical activity, dietary habit, and nutritional status data.

**Results:**

Totally 318 adults participated in the study. However, only 312 (169(54.2%) males and 143(45.8%) females) were with complete information for statistical analysis. The lowest age was 18 years, the highest 65 and the mean 38.29 ± 10.88. The prevalence of HTN was 30.8% (96/312) (95% confidence interval (CI): 25.9–36.1%), 16.4% male and 14.4% female. While 45 and older age (odds ratio (OR) 7.385, 95% CI 3.563–15.306, *p* < 0.0001), obesity (OR 126.286, 95% CI 34.481–462.514, *p* < 0.0001) and overweightness (OR 16.105, 95% CI 7.024–36.927, *p* < 0.0001), ‘substantially high risk’ (> 102 cm in men and > 88 cm in female) waist circumference (OR 1.788, 95% CI 1.008–3.173, *p* = 047), light occupational physical activity (OR 12.427, 95% CI 2.891–53.410, *p* = 0.001), walking or riding a bicycle for lower than 5 days/week (OR 13.000, 95% CI 5.140–32.882, *p* < 0.0001) and lack of sport activity (OR 18.322, 95% CI 2.430–138.169, *p* = 005), smoking (OR 2.283, 95% CI 1.284–4.060, *p* = 0.005), *khat* (OR 17.390, 95% CI 6.167–49.037, *p* < 0.0001), alcohol (OR 4.005, 95% CI 2.357–6.803, *p* < 0.0001), HH size of two (OR 2.474, 95% CI 1.250–4.895, *p* = 0.009) and ≥ 3 (OR 6.889, 95% CI 2.610–18.186, *p* < 0.0001); and HTN in family history (OR 19.417, 95% CI 10.251–36.778, *p* < 0.0001) were significant predictors of HTN in the binary logistic regression analysis; none of these were so in the multivariable model.

**Conclusion:**

Although there was a high prevalence of HTN in the study area, its independent significant predictors were not identified.

## Background

The World Health Organization (WHO) defines hypertension (HTN), also known as high or raised blood pressure (BP), as ‘a condition in which the blood vessels have persistently raised pressure’ [1]. HTN is currently an expanding global health threat. The number of hypertensive adults increased from 594 million in 1975 to 1.13 billion in 2015 globally [2]. HTN prevalence is projected to reach 29.2% by 2025 compared to the figure (26.4%) in 2000 and the number of adults with HTN in 2025 was predicted to increase by about 60.0% to a total of 1.56 billion [3]. HTN is a major risk factor for cardiovascular and chronic kidney diseases [4]. As a result, the number of deaths due to HTN is more than 9 million each year globally [5].

Lifestyle changes related to urbanization and income rise are fueling a sharp rise in chronic non-communicable diseases, including HTN, which are now becoming the leading cause of death in many low- and middle-income countries where the problem was little appreciated before. This is a double burden on acquired immunodeficiency syndrome (AIDS), tuberculosis (TB), malaria, diarrheal diseases, trachoma, etc. in these countries. In 2010, 31.5% of hypertensive cases occurred in these countries showing 7.7% age-standardized prevalence increase since 2000 [6]. This same report [6] indicated that in 2010, compared to the baseline in 2000, the level of awareness (32.3% versus 37.9%) and treatment (24.9% versus 29.0%) increased little, and control (8.4% versus 7.7%) even slightly decreased in low- and middle-income countries.

Nevertheless, there is variability in the prevalence of HTN in low-income countries. Among the Latin American population, it is 35.0%, and 20.0–30.0% in Chinese and Indian populations [3]. In sub-Saharan Africa (SSA) HTN is becoming a major health threat in an unexpected manner estimated to have a prevalence of 16.2% with 74.7 million hypertensive individuals [7, 8]. In the World Health Assembly in 2013 a plan was set to lower the prevalence of HTN by 25.0% compared with its 2010 level by 2025 [9]. To achieve this, goal collaborative efforts are urgently needed. Proactive public health interventions at the population level need to be introduced.

Because of differences in the rate of urbanization and resulting lifestyle changes among other factors are possible reasons for this variability in HTN prevalence among different populations. The main challenge in the management of HTN is a lack of awareness calling for scaling up of awareness efforts, studies of risk factors, and understanding of the impact of lifestyle changes in a contextual manner. The influence of dietary pattern, nutritional status, and lifestyle factors, as well as environmental parameters on BP is documented by various reports [10–13]. HTN predictors in high income-countries may perform differently in low- and middle-income countries due to the co-occurrence of chronic diseases, obesity and malnutrition, and other common infectious as well as noninfectious health challenges.

South African countries such as the Republic of South Africa and Namibia have the highest levels of HTN globally. A recent study in the Republic of South Africa recorded HTN prevalence of 43.0% and the study concluded that only 1 in 4 adults had normal BP [14]. This same study reported that the waist-to-height ratio (WHtR) > 0.5 and diabetes comorbidity were the most significant predictors of HTN presence. Among Namibian adults, the age-standardized prevalence of HTN was 46.0% and older age, urban residence, and being either overweight or obese were positively significantly associated with the odds of HTN [15].

In West Africa, analysis of data derived from the 2014 Ghana Demographic and Health Survey found an overall prevalence of HTN history among Ghanaian men was 5.7% [16]. Higher risk of HTN history was observed among respondents in older age groups, men with higher education and men who were widowed/divorced/separated, non-working men and men who did not smoke.

In the Middle East, the prevalence of HTN is similarly alarming. For instance, the prevalence of HTN in Oman was about 40.0% and old age, male gender, smoking, impaired glucose tolerance and body mass index (BMI) were incriminated as important covariates of HTN [17]. The authors indicated that overweight and obese had five times more likelihood of HTN than normal weight people. Less educated people, smokers and males were at a higher risk of HTN than non-smokers and females in multivariable analysis. The overall prevalence of HTN among South Asian adult (≥18 years) immigrant males, including Indian, Pakistani and Bangladeshi nationalities in the United Arab Emirates found to be 30.5% [18]. The study found that hypertensive participants were more likely to be overweight, obese, have central obesity; have a family history of HTN and were less likely to walk 30 min daily.

Similarly, a community-based cross-sectional study in India recorded the overall prevalence of HTN 17.4% with 35.0% pre-hypertensive participants and, higher age, abnormal waist circumference (WC) (≥90 cm in males and ≥ 80 cm in females), positive family history of HTN and above-average daily salt intake were significant predictors of HTN [19]. A larger study that further investigated the burden and predictors of HTN in India reported that out of 6120 participats, 43.5% HTN and predictors of a higher prevalence of HTN were older age ≥ 40 years, BMI of ≥23 kg/m^2^, larger WC, working in sedentary occupation, having diabetes mellitus, having proteinuria, and increased serum creatinine [20].

In high-income countries like Australia, HTN among children (5–17 years) reaches as high as 12.6% BMI being the strongest predictor of BP followed by socio-economic status [21]. A survey conducted on Filipino Americans in the New York City area found that 53.0% of the participants were hypertensive and older age, male gender, a BMI > 23.0 kg/m^2^, an elevated glucose reading, a family history of HTN were predictors of HTN [22]. A review of existing systematic reviews and a dual independent review of 19,309 abstracts by the US Agency for Healthcare Research and Quality [23] reported that HTN incidence increased as much as two- to four-fold between a younger (ages 18 to 40/45 years) and older (ages 40/45 to 60/65) age group, respectively although the study suggested most of the cases might have been false-positives for various reasons. Incidence was generally higher in men than women, especially men in younger populations. While incidence was also two-fold higher in overweight persons and three-fold higher in obese persons compared with those of normal weight, it was not increased in smokers compared with nonsmokers or former smokers as the study revealed. African Americans had a consistently higher incidence of HTN at rescreening than white participants as the study revealed.

The Ethiopian government’s commitment to improving health outcomes on infectious diseases such as AIDS, TB and malaria must also urgently address chronic non-communicable diseases like HTN. Even if there are a few studies in certain localities in the country [24–26], the results cannot be generalized for other settings like northeast Ethiopia. For instance, a striking variation in HTN prevalence was found across provinces in South Africa [27] suggesting temporal and spatial variations in the distribution and magnitude of the risk factors calling for more specific studies for targeted management of HTN. Up-to-date and setting-specific evidence on HTN is required. If HTN is not urgently addressed in a locality its economic and social impacts will be enormous.

The purpose of this study was, therefore, to estimate the prevalence of HTN and associated risk factors among adults (≥18 years) living in Kombolcha town and suburbs, northeast Ethiopia. The role of socio-economic and demographic, anthropometric [BMI, WC, waist-to-hip ratio (WHR), WHtR], and lifestyle factors [diet, physical activity, alcohol, etc.) are little addressed in Kombolcha which is among rapidly emerging industrial zones in the northeast of the country. With increasing urbanization, lifestyle and dietary habits are also being changed and regular monitoring of these changes in relation to the threat of HTN is necessary. It is anticipated that the outcome of the study will help health policymakers and administrators develop comprehensive and appropriate community-based health promotion strategies to encourage healthy lifestyles among its population, detect cases early enough, and choose appropriate intervention programmes.

## Methods

### Study area

The study area was Kombolcha town which is situated at 11°5′N 39°44′E latitude and 11.083°N 39.733°E longitude in northeast Ethiopia with an elevation between 1842 and 1915 m above sea level. Kombolcha is 375 km to the north of Addis Ababa. It is bordered by K’alu District in the east and south, Dessie Zuria District in the west and southwest, Tehulederie District in the north and Dessie City in the northwest. According to the Kombolcha Meteorological Agency, Kombolcha town is characterized by an average annual rainfall of 1030 mm and temperature 20.1 °C. The town is subdivided into 11 small units named *kebeles* or villages (6 urban and 5 suburban) having an area of 124.5km^2^ (12,450 ha). Based on the 2014 Ethiopian Central Statistical Agency population projection [28], the town had a total population estimate of 115,000 of whom 55,968 were men and 59,033 women (68.76% urban and 31.24% suburban inhabitants).

### Study design and participants

A multistage sampling strategy was used to select study participants. Therefore, all adult residents of the study area formed the target population. Out of the total 11 *kebeles* (urban 6 suburban 5), 5(3 urban (Kut’eba, Shewaber, Piassa) and 2 suburban (Abakolba, Fatoager)) were randomly selected (lottery method) first by clustering urban and suburban *kebeles* and taking into account the population size of each. Study households (HHs) were selected randomly from the selected *kebeles* and finally, individual adults were in turn randomly picked up from the selected HHs. Pregnant women, people with diabetes, age <18 years, those who lived in the study area for <6 months, seriously ill, or homeless were excluded from the study. The study period was from December 2016 to May 2017.

### Sample size

The sample size was calculated using open EPITools epidemiological calculator [29] using single population proportion formula, $\mathrm{n}={\mathrm{Z}}^2\times \frac{\mathrm{P}\left(1-\mathrm{P}\right)}{{\mathrm{m}}^{\mathrm{Z}}}$ where ‘Z’ is standard normal distribution curve value for the 95% confidence interval (CI) having a value of 1.96 and ‘m’ 4% margin of error (0.04). Since there was no previous study to estimate the prevalence (p) of HTN in Kombolcha a study in a nearby setting (Gondar) which recorded 13.3% at 95% CI [30] was taken. A sample size of 318 was determined considering a non-response rate of 10.0%.

### Data collection and procedure

#### Physical measurements

Anthropometric measurements were obtained from the participants wearing light clothing and no footwear. Bodyweight was measured to the nearest 0.1 kg using United Nations Children’s Fund (UNICEF) Seca digital weighing scale and height to the nearest 0.1 cm in a standing position using seca Germany a portable stadiometer. A constant tension tape was used to measure WC to the nearest 0.1 cm putting the tape at the level of the midpoint between the inferior margin of the last rib and the iliac crest in the mid-axillaries line directly over the skin or over light clothing. Waist and hip circumferences were measured with a flexible steel metric tape at the nearest 0.5 cm.

BP was measured using aneroid sphygmomanometer (Omron Healthcare Inc., Bannockburn IL, USA) on the right hand at sitting position taking three measurements with intervals of 3 min between consecutive measurements. In addition, participants were asked whether they were taking any medications for the treatment of HTN. Average systolic BP (SBP) and diastolic BP (DBP) were determined from the second and third measurements.

#### Questionnaire

A pretested structured questionnaire was used to collect data on socio-demographics, level of exercise (activity at work, travel to and from the place, recreation activities, time spent on a typical day, sport), lifestyle factors (alcohol use, khat (*Catha edulis)* chewing, salt use) and dietary habit. The 24-hour dietary recall and dietary record methods [31] were used for dietary assessment. Specifically, it was done through subjective measure using an open-ended questionnaire that was self-administered (for literate participants) and administered by a trained interviewer for illiterates. A scale of ten food groups was used in assessing the dietary diversity of patients. Using information collected from the 24-h dietary recall, the dietary diversity scores (DDSs) for individuals were derived using the Food and Agriculture Organization (FAO) guidelines for measuring HH and individual dietary diversity [32]. The dietary diversity was assessed based on the number of food groups consumed over the immediate past 24 h. A point was awarded to each food group consumed over the reference period, and the sums of all points were calculated for the DDS for each individual. The classification terciles of DDS was obtained from the 10 food groups recommended by FAO. A scale was established for this distribution: low (1–5), and high (6–10).

The questionnaire was prepared in accordance with the WHO-STEPs instrument for chronic disease risk surveillance and the Global Physical Activity Questionnaire (GPAQ) Analysis Guide and Guidelines for Measuring Household and Individual Dietary Diversity [33].

### Data quality control

The English questionnaire was translated into Amharic (participant language) for fieldwork and back to English for checking language consistency. Data was collected by the house-to-house visit. BP measurements were done by local health professionals. A digital weighing scale was used to reduce frequent calibration. The collected information was reviewed on a daily basis and errors returned to the data collectors for correction.

### Data management and analysis

Data was entered and statistical analysis performed using IBM SPSS Statistics 24 (IBM Corp, Chicago, USA). Data analysis was performed by the Chi-squared test to determine the association of anthropometric measurements and BP with gender. Further, binary and multivariable logistic regression models were used to test the association between HTN and the independent variables (socio-demographic, dietary factors, exercise level, and anthropometric measurements). Odds ratios (OR) were calculated with 95% CI. A *p*-value of less than 0.05 was considered statistically significant.

The multivariable model was constructed by forwarding the stepwise logistic regression analysis. The cutoff for retention of a variable in the model was set at *p* ≤ 0.1. All significant variables in the binary test entered in the model and explanatory variables were sequentially removed when the exit cutoff is satisfied. Age, sex and sedentary lifestyle were kept in all multivariable models, irrespective of statistical significance, to control for any confounding effects.

### Operational definitions

**HTN** is defined clinically as SBP 140 mmHg or greater or DBP 90 mmHg or greater averaged over two or more readings on two or more visits following an initial screening [34].

**Pre-hypertension:** BP readings with a systolic pressure 120-139 mmHg or a diastolic pressure 80-89 mmHg [34].

**HTN:** Readings equal to or greater than 140/90 mmHg [34].

**BMI (kg/m**^**2**^**):** 18.5: underweight; 18.5–25: normal weight; 25–30: overweight; > 30: obese [35].

**Abdominal obesity:** It is an accelerated and excessive accumulation of fat around the abdomen and stomach that may lead to health impairment [36].

**WC:** High risk ≥94 cm in men and 80 cm in women; substantially high risk > 102 cm in men and > 88 cm in women [37].

**WHR:** Substantially high ≥ 0.90 cm in men and ≥ 0.85 cm in women [37].

**WHtR:** Values for both sexes: low risk 0.40–0.499, high risk 0.50–0.59 and substantially high risk ≥ 0.60 [38].

## Results

### Socio-demography

A total of 318 respondents participated in this study with a response rate of 100%. But, only 312 (169 males and 143 females) had complete data for statistical analysis. The rest six had some missing variables and thus were excluded. Regarding socio-demographic characteristics, the male-to-female ratio was 1.18 with 54.2% males and 45.8% females. The age of the study population ranges from 18 to 65 years and the mean 38.29 ± 10.88. Hundred forty respondents (44.9%) were 31–45 years old. Hundred thirty-six (43.6%) were merchants followed by 134(42.9%) civil servants and the rest 42(13.5%) farmers. Hundred ninety-one (61.2%) participants were married. While 202(64.7%) participants were grade 8 or 10 complete those having qualification of certificate and above were 110(35.3%). Twenty-eight (8.9%) participants were living alone, 210(67.3%) living in HH size of two and the rest 74(23.7%) were from HH size of three and above. Hundred eighty-four (58.9%) participants had a history of HTN and other related diseases among their family members.

### DDS

All the major types of food groups are available in the local market year-round. When asked, 99.0% of the participants reported that they had consumed cereals, pulses, condiments, and 96.2% had used sugars and fast foods. Oil/fats; milk and milk products; meat, fish and poultry; and fruits were consumed by 43.3, 38.1, 33.7, and 26.0% of the respondents respectively. The mean DDS was 6.5 ± 1.9. Overall, 169(54.2%) had low DDS and the rest 143(45.8%) had high.

### Nutritional status

The BMI of the respondents ranged from 18.21 to 36.94 kg/m^2^, with a mean of 25.7 ± 2.4 kg/m^2^. The mean BMI of the male respondents was 23.5 ± 3.3 kg/m^2^ whereas that of the females was 24.1 ± 4.4 kg/m^2^ (Table 1). Overall, 143(45.8%) of the participants had BMI < 25.0 kg/m^2^, and 169(54.2%) had ≥ 25.0 kg/m^2^. Hundred thirty-nine (44.6%) participants were overweight and only 30(9.6%) obese. In addition, slightly higher proportions of females than males were overweight (46.2% versus 43.2%), and a higher proportion of females were obese (13.3% females versus 6.5% males). Categorized by sex, abdominal obesity occurred among 32.2% females and 24.8% males.

**Table 1:** Gender-based anthropometric and BP measurement for the study participants as tested by the Chi-squared test

Variable	Alternatives	Sex^a^		Total, n (%)	*P*-value
		Male, n (%) Female, n (%)			
Abdominal obesity	Present	42 (24.9)	46 (32.2)	88 (28.2)	0.018**
	Absent	127 (75.2)	97 (67.8)	224 (71.8)	
BMI	Normal	85 (59.4)	58 (40.6)	143 (45.8)	<0.0001**
	Overweight	73 (52.5)	66 (47.5)	139 (44.6)	
	Obese	11 (36.7)	19 (63.5)	30 (9.6)	
WC	*Low risk*	82 (61.7)	51 (38.3)	133 (42.6)	0.478
	*Increased risk*	37 (42.5)	50 (57.5)	87 (27.9)	
	*Substantially increased risk*	50 (54.3)	42 (45.7)	92 (29.5)	
WHR	*Low risk*	42 (30.0)	98 (70.0)	140 (44.9)	0.075
	*Substantially increased risk*	127 (73.8)	45 (26.2)	172 (55.0)	
WHtR	*Low risk*	22 (13.1)	42 (29.6)	64 (20.6)	0.138
	*Increased risk*	88 (52.4)	77 (54.2)	165 (53.2)	
	*Substantially increased risk*	59 (34.9)	23 (16.2)	81 (26.2)	
SBP	Normal	99 (58.6)	78 (54.5)	177 (56.7)	<0.0001**
	Pre-HTN	34 (20.1)	34 (23.8)	68 (21.8)	
	HTN	36 (21.3)	31 (21.7)	67 (21.5)	
DBP	Normal	127 (75.1)	102 (71.3)	229 (73.4)	<0.0001**
	Pre-HTN	10 (5.9)	15 (10.5)	25 (8.0)	
	HTN	32 (18.9)	26 (18.9)	58 (18.6)	

^a^The sex-adjusted anthropometric and BP measurements cutoff values were considered for each group, *BP* Blood pressure, *DBP* Diastolic blood pressure, *SBP* Systolic blood pressure, *BMI* Body mass index, *WC* Waist circumference, *WHR* Waist-to-hip ratio, *WHtR* Waist-to-height ratio, *n* Number, **statistically significant, %: percentage

### Physical activity level and sedentary lifestyle

Only 34(10.9%) of the participants reported that their occupational physical activity was of the *vigorous*-*intensity* type followed by 95(30.4%) *moderate* and 183(58.7%) *light* at least for 20 min continuously per week. Two-hundred twelve (67.9%) respondents reported no sport, 76(24.3%) do a *moderate-intensity* type and the rest 24(7.7%) practice *vigorous-intensity* sport. Similarly, 32(10.3%) and 280(89.7%) participants walk or ride a bicycle for 1–4 and 5–7 days (for 20 min each day) per week, respectively. The durations of sedentary activity on a typical day were > 120, 60–120 and < 60 min for 253(81.1%), 51(16.3%) and 8(2.6%) participants respectively (Table 2).

**Table 2:** Binary logistic regression analysis of HTN by socio-demography, anthropometry, dietary pattern, lifestyle and nutritional status of adults in Kombolcha

Variable	n (%)	HTN, n (%)	Binary analysis		
			COR	(95% CI)	*P*-value
Sex					
Female (Ref)	143 (45.8)	45 (31.4)	1.000	–	
Male	169 (54.2)	51 (30.1)	0.941	0. 581–1.524	0.806
Age (year)					
18–30 (Ref)	85 (27.2)	13 (15.2)	1.000	–	
31–45	140 (44.9)	35 (25.0)	1.897	0.940–3.831	0.074
≥ 46	87 (27.9)	48 (55.1)	7.385	3.563–15.306	<0.0001*
BMI					
Normal (Ref)	143 (45.8)	7 (4.8)	1.000	–	
Overweight	139 (44.2)	63 (45.3)	16.105	7.024–36.927	<0.0001*
Obese	30 (9.6)	26 (86.6)	126.286	34.481–462.514	<0.0001*
WC					
Low risk (Ref)	133 (42.6)	34 (25.5)	1.000	–	
Increased risk	87 (27.9)	27 (31.0)	1.310	0. 720–2.384	0.376
Substantially increased risk	92 (29.5)	35 (38.0)	1.788	1.008–3.173	0.047
WHR					
Low risk (Ref)	140 (44.9)	41 (29.8)	1.000	–	
Substantially increased risk	172 (55.1)	55 (31.9)	1.135	0.699–1.843	0.609
WHtR					
Low risk (Ref)	66 (21.2)	19 (28.7)	1.000	–	
Increased risk	165 (52.9)	43 (26.0)	0.872	0.461–1.647	0.673
Substantially increased risk	81 (25.9)	34(41.9)	1.789	0.896–3.574	0.099
Occupational physical activity					
Vigorous (Ref)	34 (10.9)	2 (5.8)	1.000	–	
Moderate	95 (30.4)	14 (14.7)	2.765	0.595–12.862	0.195
Light	183 (58.7)	80 (43.7)	12.427	2.891–53.410	0.001*
Walking or riding a bicycle					
5–7 days (Ref)	280 (89.7)	70 (25.0)	1.000	–	
1–4 days	32 (10.3)	26 (81.2)	13.000	5.140–32.882	<0.0001*
Sport					
Vigorous (Ref)	24 (7.7)	1 (4.1)	1.000	–	
Moderate	76 (24.4)	1 (1.3)	0.307	0.018–5.098	0.410
No	212 (67.9)	94 (44.3)	18.322	2.430–138.169	<0.005*
Sedentary lifestyle (minutes/day)					
<60 (Ref)	8 (2.6)	0 (0.0)	1.000	–	
61–120	51 (16.3)	4 (7.8)	137,489,063.300	0.000-	0.999
> 121	253 (81.1)	92 (36.4)	923,140,853.600	0.000-	0.999
Cigarette smoking					
No (Ref)	251 (80.4)	68 (27.1)	1.000	–	
Yes	61 (19.6)	28 (45.9)	2.283	1.284–4.060	0.005*
Khat chewing					
No (Ref)	97 (31.1)	4 (4.1)	1.000	–	
Yes	215 (68.9)	92 (42.8)	17.390	6.167–49.037	< 0.0001*
Binge drinking					
No (Ref)	228 (73.1)	51(22.4)	1.000	–	
Yes	84 (26.9)	45(53.6)	4.005	2.357–6.807	<0.0001
Resident					
Suburban (Ref)	82 (26.3)	25 (30.5)	1.000	–	
Urban	230 (73.7)	71 (30.9)	1.018	0.589–1.760	0.949
Job					
Farmer (Ref)	42(13.5)	11(26.2)	1.000	–	
Merchant	136 (43.6)	41 (30.1)	1.216	0.558–2.651	0.622
Civil servant	134 (42.9)	44 (32.8)	1.378	0.634–2.995	0.419
Marital status					
Divorced (Ref)	54 (17.3)	14 (25.9)	1.000	–	
Unmarried	67 (21.5)	21 (31.3)	1.304	0.587–2.897	0.514
Married	191 (61.2)	61(31.9)	1.341	0.679–2.647	0.398
Education					
Grade 8 complete (Ref)	132 (42.3)	37 (28.0)	1.000	–	
Grade 10 complete	70 (22.4)	21 (30.0)	1.100	0.582–2.080	0.786
Certificate and above	110 (35.3)	38 (34.5)	1.355	0.785–2.341	0.276
Household size					
Three and above (Ref)	74 (23.7)	12 (16.2)	1.000	–	
Single	28 (8.9)	15 (53.6)	2.474	1.250–4.895	0.009*
Two	210 (67.3)	68 (32.3)	6.889	2.610–18.186	<0.0001*
Family history of HTN					
No (Ref)	128 (41.1)	81 (63.3)	1.000	–	
Yes	184 (58.9)	15 (8.1)	19.417	10.251–36.778	<0.0001*
DDS					
High (Ref)	143 (45.8)	45 (31.5)	1.000	–	
Low	169 (54.2)	51 (30.2)	0.941	0.581–1.524	0.806

*DDS* Dietary diversity score, *BMI* Body mass index, *WC* Waist circumference, *WHR* Waist-to-hip ratio, *WHtR* Waist-to-height ratio, *n* Number of people, *COR* Crude odds ratio, *CI* Confidence interval, *statistically significant, %: percentage

### Behavioral characteristics

While 61(19.6%) participants reported that they smoke cigarettes regularly during the study period and prior, 251(80.4%) did not ever smoke. Similarly, 215(68.9%) chew *khat* (*Catha edulis*) continuously for extended hours daily and 97(31.1%) responded negatively. Two-hundred twenty-eight (73.1%) participants were no-users of alcoholic drinks and 84(26.9%) answered in the affirmative. Three-hundred (96.2%) individuals use salt in their diet and only 12(3.8%) avoid.

### Anthropometric and BP measurements

While 139(44.5%) participants were overweight, the prevalence of obesity was 9.6% (30/312). Concerning the WC, 92(29.4%) were in the category of *substantially increased risk*, 87(27.8%) *increased risk* and 133(42.6%) at *low risk* for HTN as per the WHO categorization. Based on WHR, 172(55.1%) of the individuals were under *increased risk* for developing cardiovascular diseases. Risk classification with WHtR showed that 165(52.8%) and 81(25.9%) participants had *high risk* and *substantially increased risk*, respectively.

### Prevalence of HTN

The mean SBP of the study population was 129.6 ± 13.7 mmHg and that of DBP was 83.9 ± 8.2 mmHg. A total of 96(30.7%) individuals were hypertensive at 95% CI. Analysis of BP by age showed that the mean BP (both systolic and diastolic) was highest in the age group ≥45 years and the prevalence of HTN generally increased with age. HTN prevalence in males was 30.2% (51/169) and among females 31.5% (45/143) with no statistically significant difference (Table 2). There was some variation in the prevalence of SBP and DBP, 67(21.4%) participants had systolic HTN and 58(18.5%) diastolic HTN.

Age of 45 and older (OR) 7.385, 95% CI 3.563–15.306, *p* < 0.0001), obesity (OR 126.286, 95% CI 34.481–462.514, *p* < 0.0001) and overweightness (OR 16.105, 95% CI 7.024–36.927, *p* < 0.0001), *substantially high risk* (> 102 cm in men and > 88 cm in female) WC (OR 1.788, 95% CI 1.008–3.173, *p* = 047), *light occupational physical activity* (OR 12.427, 95% CI 2.891–53.410, *p* = 0.001), walking or riding a bicycle for lower than 5 days/week (OR 13.000, 95% CI 5.140–32.882, *p* < 0.0001) and lack of sport activity (OR 18.322, 95% CI 2.430–138.169, *p* = 005), smoking (OR 2.283, 95% CI 1.284–4.060, *p* = 0.005), *khat* chewing (OR 17.390, 95% CI 6.167–49.037, *p* < 0.0001), alcohol drinking (OR 4.005, 95% CI 2.357–6.803, *p* < 0.0001), HH size of two (OR 2.474, 95% CI 1.250–4.895, *p* = 0.009) and ≥ 3 (OR 6.889, 95% CI 2.610–18.186, *p* < 0.0001), and HTN in family history (OR 19.417, 95% CI 10.251–36.778, *p* < 0.0001) were significant predictors of HTN in the binary logistic regression analysis (Table 2). But, none of these variables was significantly associated with HTN in the multivariable model.

## Discussion

The prevalence of HTN in the present study is 30.8% which is comparable to previous similar community-based studies in Ethiopia. Reports from Addis Ababa [39] and Gondar, northwest Ethiopia [30] showed 30.2 and 28.3% HTN prevalence respectively. Further, the present result is comparable with results from other SSA countries. For instance, 31.0% prevalence of HTN was reported in rural and urban populations of Abia State, Nigeria [40].

On the other hand, the prevalence of HTN in this study was higher than what was obtained from Bahir Dar (25.1%) in northwest Ethiopia [25]. A study that assessed the relationship between socio-economic status and HTN among teachers and bankers in Addis Ababa found HTN prevalence of 19.1 and 21.8% for bankers and teachers, respectively [41]. Reports from southern Ethiopia [42] found HTN prevalence of 9.9%. Discrepancies in HTN reports could be because of differences in lifestyle behaviors and other related factors.

The 30.2 and 31.5% proportion of hypertensive male and female participants, respectively, is comparable with other studies like Tesfaye et al. 2009 [39] who reported 31.5% for men and 28.9% for women in Addis Ababa. A meta-analysis report for Ethiopia shows that the prevalence of HTN among males was 20.6% and females 19.2% [43]. These same authors in their systematic review targeting the literature between January 2000 and April 2015 demonstrated that the rate of HTN prevalence in Ethiopia varied widely, with the highest rate of 31.5% in males and the lowest (0.8%) in females overall between 20.0–30.0%.

In this study, the prevalence of overweight was 44.6% out of which 52.5% male and 47.5% female and overall obesity 9.6% (36.7% male and 63.5% female). This finding shows a considerably high prevalence of overweight and obesity among adults in Kombolcha. Two thousand eleven Ethiopian Demographic and Health Survey [44] showed that women in Addis Ababa had the highest prevalence of overweight (22.2%) across the country. However, this figure was slightly lower than our estimate of 47.5%. Out of 44.6% overweight adults of Kombolcha, 45.3% were HTN. From the 9.6% obese, 86.7% were positive for HTN. Overweight and obesity, including abdominal obesity, were more prevalent among females, while elevated BP was high in both males and females.

Most participants (81.1%) practiced sedentary lifestyle (television watching, game playing, *khat* chewing) demonstrating that most of the adult population has a sedentary lifestyle, out of this 36.4% were hypertensive. These can be explained by modernization which is characterized by the increasing use of motorized transport and more sedentary lifestyles**.**

Walking or riding a bicycle 1–4 days/week was found to be significantly associated with the odds of HTN, although it is not when adjusted for confounding factors, which is in agreement with a similar study from Gilgel Gibe in southwest Ethiopia [45]. This finding might be explained by the decreased energy expenditure among this group. Sport activity was positively associated with the odds of HTN, gain without adjustment. Participants who cannot do sport were exposed 18 times for HTN than their counterparts probably due to the accumulation of lipids in blood vessels that could damage blood vessels as well as heart muscles.

In general, the study found a high prevalence of HTN along with increased prevalence of overweight and obesity which are high-risk factors for developing other cardiovascular disorders as well. If corrective actions are not taken, there will be intolerable health consequences among the population in the recent future. Therefore, the health system needs to adopt regular screening and diagnostic services. Sustainable interventions require awareness creation so that high-risk groups and the general population could save themselves. Weight loss, dietary reduction, change in lifestyle, and increased physical activity need to be promoted even if the current study failed to detect any significant association between these variables in the multivariable model.

This study has certain limitations. It was a restricted population survey with a relatively small sample size which could have inherent sampling bias making it difficult to generalize the findings for Kombolcha residents as a whole. The particularly very small and disproportionate (incomparable) sample size for different categories of some variables might have impacted the statistical analysis of the multivariable model. Further, the study involves only anthropometric and BP measurements without biochemical tests due to cost, time and other related constraints. The women and even some males might not self-report their alcohol-drinking and smoking habits which are socially undesirable behaviors within the community.

## Conclusion

The data demonstrated an alarming rate of increasing weight, raised BP, widespread physical inactivity and sedentary behavior among adults although these factors were not independently significantly associated with HTN probably for inherent sampling problems. The findings emphasize the need for adopting a health policy to further assess the magnitude of HTN which typically does not cause symptoms, and devise control strategies in the study area.

## Data Availability

The datasets used and/or analysed during the current study available from the corresponding author on reasonable request.

## Abbreviations

AIDS: Acquired immunodeficiency syndrome
BMI: Body mass index
CI: Confidence interval
DBP: Diastolic pressure
DDS: Dietary diversity score
FAO: Food and agriculture organization
GPAQ: Global Physical Activity Questionnaire
HHs: Households
HTN: Hypertension
P: Prevalence
SBP: Systolic blood pressure
SD: Standard deviation
TB: Tuberculosis
UNICEF: United Nations international Children’s emergency fund
WC: Waist circumference
WHO: World Health Organization
WHR: Waist-to-hip ratio
WHtR: Waist-to-height ratio
